# Wearable device for continuous sweat lactate monitoring in sports: a narrative review

**DOI:** 10.3389/fphys.2024.1376801

**Published:** 2024-04-04

**Authors:** Geonwoo Yang, Junggi Hong, Seung-Bo Park

**Affiliations:** Graduate School of Sports Medicine, CHA University, Seongnam-si, Republic of Korea

**Keywords:** wearable devices, sweat lactate, sports performance, continuous monitoring, biosensors

## Abstract

In sports science, the use of wearable technology has facilitated the development of new approaches for tracking and assessing athletes’ performance. This narrative review rigorously explores the evolution and contemporary state of wearable devices specifically engineered for continuously monitoring lactate levels in sweat, an essential biomarker for appraising endurance performance. Lactate threshold tests have traditionally been integral in tailoring training intensity for athletes, but these tests have relied on invasive blood tests that are impractical outside a laboratory setting. The transition to noninvasive, real-time monitoring through wearable technology introduces an innovative approach, facilitating continuous assessment without the constraints inherent in traditional methodologies. We selected 34 products from a pool of 246 articles found through a meticulous search of articles published up to January 2024 in renowned databases: PubMed, Web of Science, and ScienceDirect. We used keywords such as “sweat lactate monitoring,” “continuous lactate monitoring,” and “wearable devices.” The findings underscore the capabilities of noninvasive sweat lactate monitoring technologies to conduct long-term assessments over a broad range of 0–100 mM, providing a safer alternative with minimal infection risks. By enabling real-time evaluations of the lactate threshold (LT) and maximal lactate steady state (MLSS), these technologies offer athletes various device options tailored to their specific sports and preferences. This review explores the mechanisms of currently available lactate monitoring technologies, focusing on electrochemical sensors that have undergone extensive research and show promise for commercialization. These sensors employ amperometric reactions to quantify lactate levels and detect changes resulting from enzymatic activities. In contrast, colorimetric sensors offer a more straightforward and user-friendly approach by displaying lactate concentrations through color alterations. Despite significant advancements, the relationship between sweat lactate and blood lactate levels remains intricate owing to various factors such as environmental conditions and the lag between exercise initiation and sweating. Furthermore, there is a marked gap in research on sweat lactate compared to blood lactate across various sports disciplines. This review highlights the need for further research to address these shortcomings and substantiate the performance of lactate sweat monitoring technologies in a broader spectrum of sports environments. The tremendous potential of these technologies to supplant invasive blood lactate tests and pioneer new avenues for athlete management and performance optimization in real-world settings heralds a promising future for integrating sports science and wearable technology.

## 1 Introduction

In contemporary sports, gaining a competitive edge hinges on the precise understanding and vigilant monitoring of athletes’ physiological states. The ability to track physiological changes in real-time during sports is paramount to sustaining optimal athletic performance (Mujika, 2017). Recent advancements in wireless sensors and wearable technology have revolutionized the measurement and interpretation of key physical markers. Among these, lactate is a crucial indicator used for assessing physiological reactions in the body. Monitoring lactate levels is especially significant, as it provides insights into an athlete’s aerobic and anaerobic capacities that provide valuable insights for developing tailored training and recovery strategies (Goodwin et al., 2007; Buono et al., 2010; Casado et al., 2022). The balance between lactate production and elimination in tissues affects blood lactate concentration (Stallknecht et al., 1998). This balance fluctuates even with minor, short-lasting changes; thus, even slight changes in the balance can have significant diagnostic implications for athletes (Brooks, 2018).

The current gold standard for lactate monitoring is invasive and episodic, requires toleration of discomfort (e.g., needle pricks, blood leakage, and potential infection risks), and also has limitations in detection speed and portability (Daboss et al., 2022; Aguilar-Torán et al., 2023). This has led to a surge in interest and demand for noninvasive monitoring technologies within sports science (Yang et al., 2022; Rabost-Garcia et al., 2023). One of the primary body fluids studied in noninvasive lactate monitoring is sweat (Van Hoovels et al., 2021). Compared to other fluids, sweat is easier to collect and less prone to contamination than blood (Xuan et al., 2023a). In light of these factors, wearable devices that offer continuous monitoring of sweat lactate levels have attracted much interest in sports.

**FIGURE 1:**
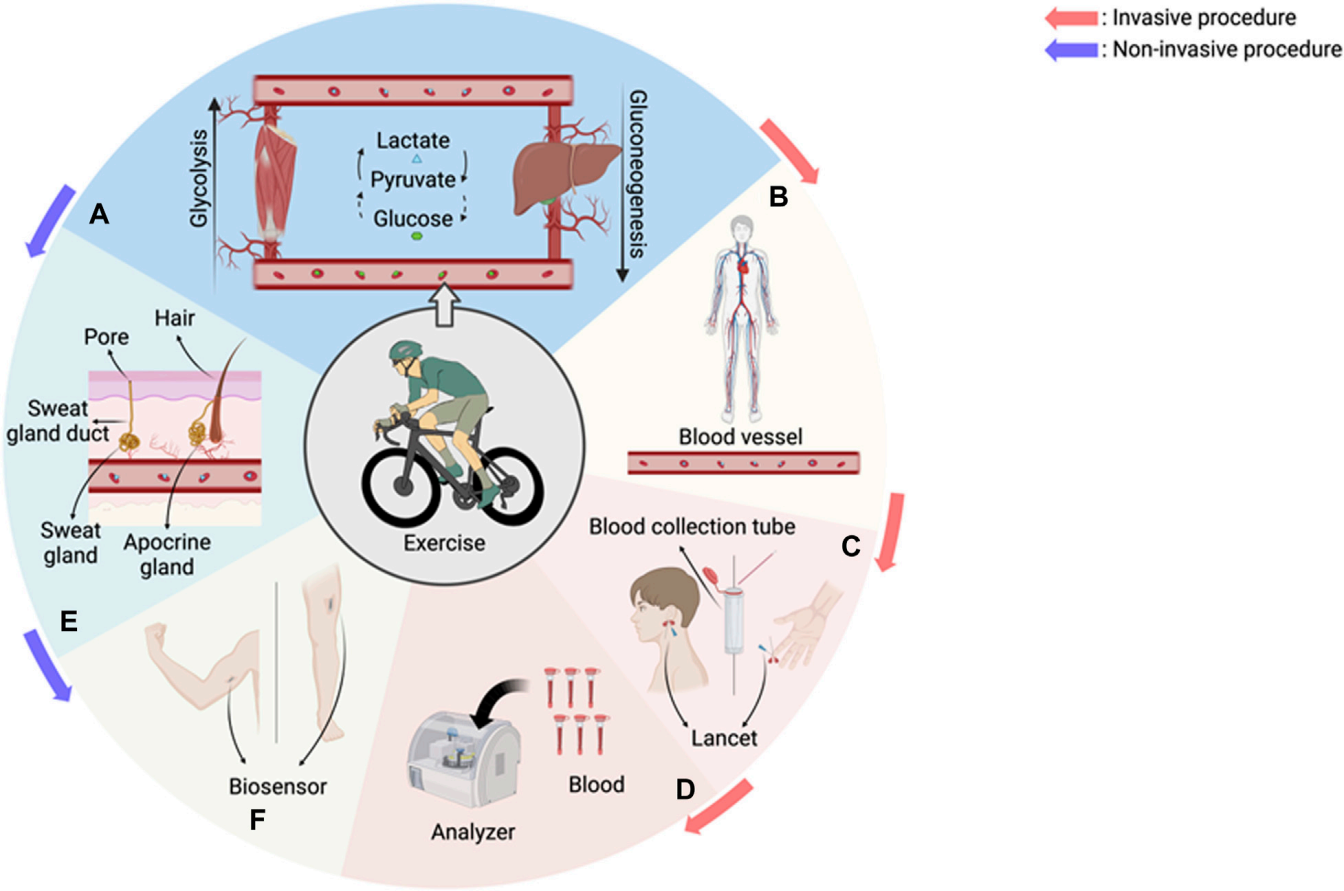
An overview of the physiological changes in our body during exercise and invasive and non-invasive monitoring technologies. **(A)** During exercise, glucose is broken down into lactate through glycolysis in the muscles, and lactate is transported to the liver, where it is resynthesized into glucose through gluconeogenesis and transported back to the muscles. **(B)** Lactate is transported through blood vessels to microscopic vessels and organs throughout the body. **(C)** The traditional method to measure lactate is through invasive blood sampling from fingertips, earlobes, etc. **(D)** After blood collection, an analyzer is used to analyze the level of lactate in the blood. Multiple blood sampling is required and is discontinuous. **(E)** Sweating system. Sweat contains substances produced by sweat glands (lactate, urea, cytokines) and substances produced by apocrine glands (lipids, proteins, sugars, ammonia) (Baker and Wolfe, 2020). **(F)** Sweating system. Sweat contains substances produced by sweat glands (lactate, urea, cytokines) and substances produced by apocrine glands (fat, protein, sugar, ammonia). Recreated with BioRender.com.

This review provides an overview of the technological advancements and potential applications of wearable devices for continuous sweat lactate monitoring currently emerging in the market (Figure 1). It specifically examines the current technological progress and potential feasibility of real-time monitoring wearable technologies in sports. This study begins by highlighting the importance of lactate in sports and its impact on elite athletes, underscoring the need for wearable devices by addressing the limitations of conventional lactate measurement methods. It also discusses the current state of technological development and industry trends and how sweat biomarker monitoring technologies can contribute to advancements in sports science. Thus, we ultimately aim to enhance the understanding of the innovative potential applications of real-time lactate monitoring wearable technologies in sports. In doing so, we seek to propose ways in which these technologies can be utilized to improve athletes’ performance and foster optimized training environments.

## 2 Literature search methods and results

This narrative review used online databases such as PubMed, Web of Science, and ScienceDirect to search for articles published between 1975 and 2024. The search incorporated a combination of terms and keywords, including “sweat lactate monitoring,” “continuous lactate monitoring,” “noninvasive,” “biosensor,” “amperometric,” “electrochemical,” “colorimetric,” and “wearable device.” We focused on original articles and reviews published in English. The titles and abstracts of the articles were reviewed to ensure the inclusion of relevant studies. After a preliminary review, full texts of the articles were reviewed; G.Y., S.-B.P. and J.H. evaluated each article to determine eligibility.

**FIGURE 2:**
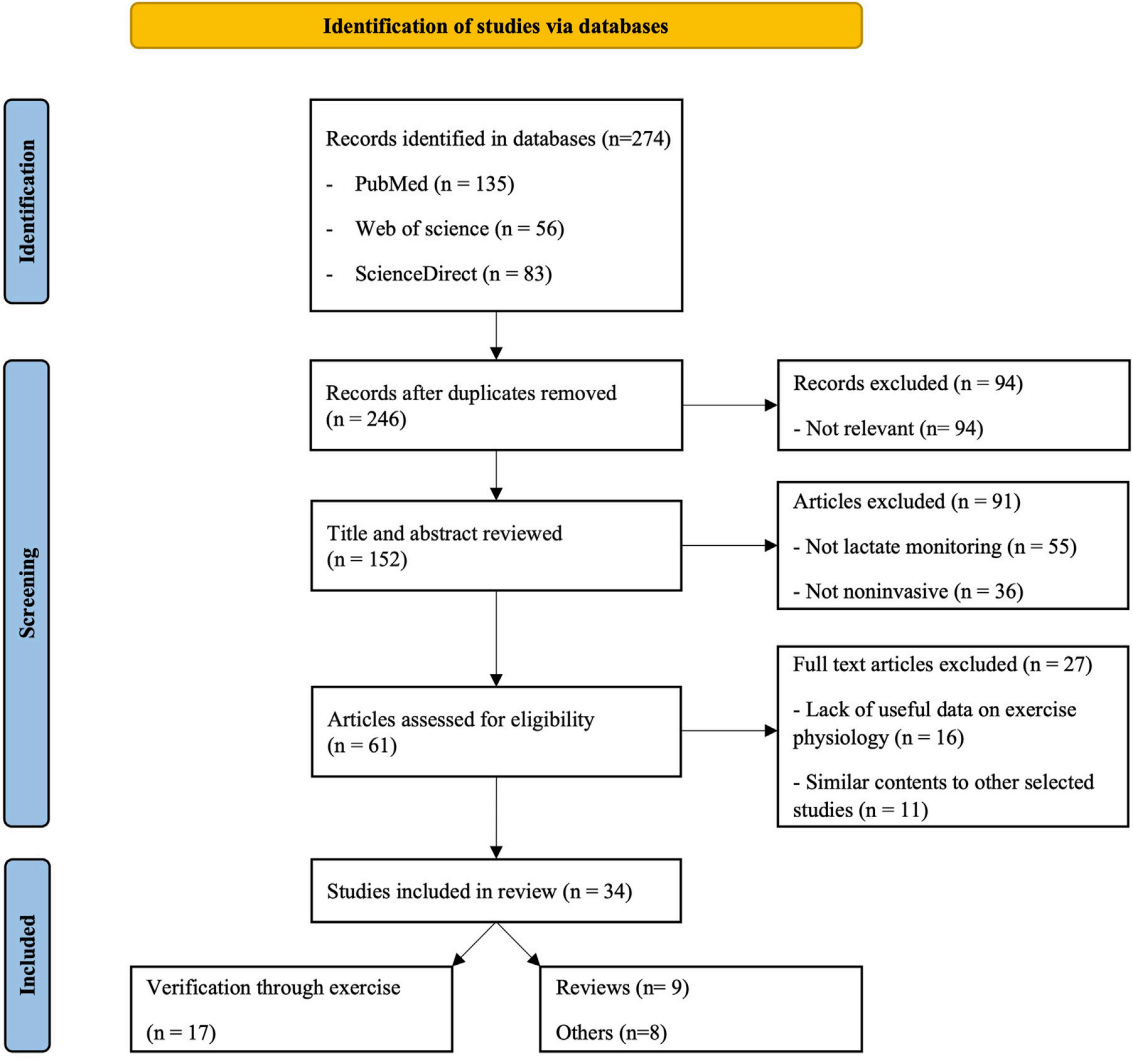
Flow chart of study selection for this review.

The flowchart of the literature search and identification of relevant articles for review are depicted in Figure 2. After the initial search, 274 articles were identified from the mentioned databases. We excluded 27 duplicate search results. Of the 246 screened articles, 94 were excluded because they were only related to one of the keywords, “sweat,” “lactate,” or “monitoring,” or lacked relevance to the core topic. Upon further review of the titles and abstracts of all selected studies, an additional 55 articles were excluded for not reporting on lactate monitoring, and 36 articles were excluded for using a technology other than the noninvasive technology. From the remaining 61 articles, 27 were excluded due to a lack of useful data related to sports or the inclusion of information similar to that reported in other screened studies, resulting in a total of 34 key articles. Of these, 17 articles that included validation of biosensors through exercise were categorized and reviewed in Table 3. We also included nine reviews to provide an overall understanding of the trends in this field, along with eight articles introducing monitoring technologies for other bodily fluids, such as “saliva” and “tears,” and those related to “continuous glucose monitoring.”

## 3 Utilization of lactate in sports

Lactate is produced by the anaerobic glycolytic system (fast glycolysis), meaning lactate metabolism is an essential pathway in physical exercise (Brooks, 2020). The concentration of lactate in the blood reflects the balance between lactate production and elimination (Maciejewski et al., 2020), which should be within the range of 0.5–2.2 mmol/L in healthy individuals (Pundir et al., 2016). Lactic acid exists in equilibrium with lactate, and the equilibrium is maintained by the body’s pH level (Schmidt et al., 2021). At the normal body pH of 7.4, lactic acid exists predominantly as lactate, which has one less hydrogen ion (Crapnell et al., 2021; Certo et al., 2022). However, in many studies, the terms “lactic acid” and “lactate” are used interchangeably (Cairns, 2006; Hall et al., 2016).

Robergs et al. (2004) reported that while ‘lactic acid’ has historically been perceived as a fatiguing substance resulting from continuous muscle contraction, recent understanding emphasizes that ‘lactate’ is, in fact, a beneficial compound capable of sustaining exercise. The mechanism underlying this phenomenon involves the simultaneous production of lactate and hydrogen ions (H^+^), where the latter contributes to metabolic acidosis. Lactate, generated through the conversion of NADH + H^+^ and pyruvate, serves as an energy source, while oxidized NAD^+^ aids in regenerating pyruvate in step 6 of glycolysis (Chandel, 2021; Luengo et al., 2021). Notably, Brooks et al. (2005) emphasized the role of lactate in improving endurance by delaying metabolic acidosis. Lactate, upon entering Type 1 muscle fibers, is utilized for energy production within mitochondria via Monocarboxylate Transporters (MCTs) (Brooks et al., 2022). Consequently, it is crucial to distinguish between ‘lactic acid’ and ‘lactate’ (Hall et al., 2016). In contrast, lactate serves as an energy source for sustained exercise, and the primary contributor to metabolic acidosis is the accumulation of H^+^ ions and Phosphate (Pi) (Woodward and Debold, 2018). By measuring glucose and lactate levels during exercise, the primary energy sources used at different performance intensities and durations can be determined (Heinonen et al., 2012; Alghannam et al., 2021). This understanding can enable trainers to design training programs that set exercise intensities to minimize unnecessary expenditure of energy sources (carbohydrates) and sustain optimal performance over extended periods (Flockhart et al., 2021; Casado et al., 2022).

This is particularly important in high-intensity, intermittent sports. Compared to speed sports where maximum anaerobic performance is key given the need to cover short distances in minimal time (Heck et al., 2003), most sports requiring repeated transitions between high- and low-intensity movements over an extended period, the ability to recover by utilizing lactate produced in the preceding exercise as an energy source (glucose) becomes a crucial step (Schünemann et al., 2023). Sports such as middle-distance rowing, cycling, and marathons require athletes to have the ability to exert higher power output during initial sprints and final spurts than the average output during the entire race. In ball sports such as soccer, American football, and hockey, players repeatedly alternate between numerous sprints and low-intensity jogging, albeit across different positions (Iaia et al., 2009). In these sports, the phosphagen and glycolytic energy systems with high flux rates need to be primarily utilized during high-intensity actions, which requires the oxidative system that contributes the most to energy metabolism during the game, to support the capacity to accommodate such intense actions (Bangsbo et al., 1990; Xu and Rhodes, 1999; Gastin, 2001; Balasekaran et al., 2023).

Repeated training using glucose and lactate data can increase the endurance of athletes, enabling them to train at high intensities without accumulating lactate in muscle tissues (Lee et al., 2021). Such training enhances energy efficiency in trained athletes by increasing the contribution of the aerobic energy system at the same exercise intensities compared to in untrained athletes (Zapata-Lamana et al., 2018; Hebisz et al., 2022). Marathon runners with high cardiopulmonary endurance minimize glucose utilization during the race and predominantly rely on fat oxidation, where lactate and fats serve as the main energy sources (Sjödin and Svedenhag, 1985; van Loon, 2004; Aengevaeren et al., 2020).

**FIGURE 3:**
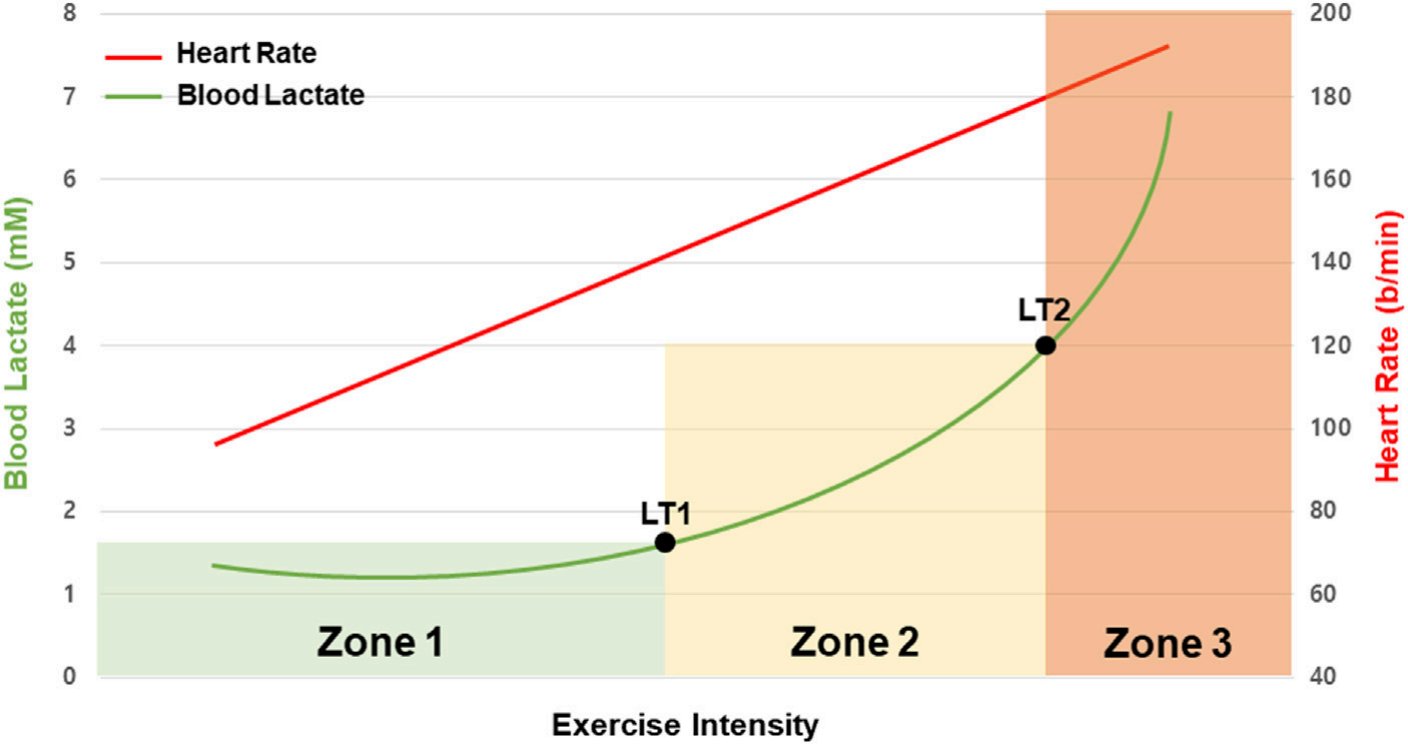
Typical blood lactate (green line) and heart rate (red line) response to the multi-stage test based on exercise intensity. The three aerobic training zones (Zones 1–3) are determined by the multi-stage test’s first (LT1) and second (LT2) lactate thresholds. The LT1 represents the rise in blood lactate above the initial value. The LT2 denotes an acceleration of blood lactate accumulation.

The lactate threshold (LT) refers to two critical points where lactate accumulation increases sharply with progressively increasing exercise intensity (e.g., speed, resistance) (Binder et al., 2008) (Figure 3). LT1 is known as the first inflection point where lactate level starts to increase, and LT2 is the exercise intensity at which the blood lactate concentration exceeds 4 mmol/L (Heck et al., 1985). Based on these two points, exercise intensity zones are defined as zone 1 (low intensity), zone 2 (moderate intensity), and zone 3 (high intensity).

In Zone 1, which is below the LT1 threshold, the body primarily relies on fats rather than carbohydrates for energy. During exercises in this intensity zone, the rate of lactate elimination effectively matches its production, so there is no marked rise in blood lactate levels even during prolonged exercise (Nordheim and Vøllestad, 1990; Schrauwen et al., 2002).

In Zone 2, which is between LT1 and LT2, there is a noticeable increase in lactate production, resulting in elevated blood lactate concentrations. This zone includes the maximal lactate steady state (MLSS), characterized by a balance between lactate production and elimination maintained for about 30 min with minimal fluctuations in lactate concentration (under 1 mmol/L) (Aunola and Rusko, 1992).

Zone 3 encompasses exercise intensities surpassing LT2. Exercise in this zone leads to a sustained increase in blood lactate levels throughout the duration of activity (Jacob et al., 2023). Typically, this zone is reached at higher exercise intensities, often exceeding 85% of $\dot{V}O_{2}\max$ (maximal oxygen uptake), at 90% of heart rate max (HRmax) and ventilatory threshold (VT) 2 (Coutts et al., 2003; Bentley et al., 2007; Plato et al., 2008).

There are several criteria for categorizing exercise intensity, including HR, $\dot{V}O_{2}\max$ , and VT, but lactate is considered one of the most sensitive biomarkers (Beneke et al., 2011; Jamnick et al., 2020). Therefore, using the LT test to gauge exercise intensity enables optimal preparation for competition, including season readiness, training periodization, and performance enhancement for elite athletes (Henritze et al., 1985).

In their study of 23 healthy participants and 42 participants with cardiovascular diseases, Seki et al. (2021) reported a correlation between sweat LT1 and blood LT1 as well as between sweat LT1 and VT1 during progressively intense cycling on a cycle ergometer. Based on these results, they recommended the potential use of real-time sweat lactate monitoring for observing LT1. In another study where elite kayakers performed submaximal and maximal self-paced tests using a kayak ergometer, the previously stable sweat lactate levels increased sharply when the blood lactate level had reached LT2. Similar results were observed for cyclists in the same study (Karpova et al., 2020).

Xuan et al. (2023a) conducted a study involving elite cyclists and triathletes. They used a cycle ergometer test while the participants increased the cycling intensity every 15 min. They observed that the sweat/blood lactate ratio that remained consistent after exercise varied between the two LT points, indicating the utility of sweat lactate monitoring in providing individualized physiological data (Okawara et al., 2023). Mao et al. (2020) reported that physiological responses measured through sweat lactate monitoring using biosensors during cycling were consistent with the ranges of MLSS in professional speed skaters.

Periodization based on the measurement of blood metabolites has limitations due to the invasive nature of such measurements, making it challenging to monitor physiological changes in athletes in real-time during training (Jia et al., 2013). As a result, monitoring during training often relies on HR even when training management is based on blood lactate levels. Therefore, the development of real-time lactate monitoring wearable devices presents a promising alternative to HR monitors, offering the potential for precise monitoring of physiological parameters. Considering the importance of training periodization not only for performance enhancement but also for injury prevention in athletes, real-time data collected during training can be used to measure training load and adjust training volume flexibly based on the predicted fatigue levels. Previous studies have primarily focused on validating the accuracy and durability of sensors. In this regard, there is a need for more field-friendly physiological protocols to enhance the credibility and applicability of developed wearable devices in real-life sports settings.

## 4 Invasive and noninvasive analytical techniques

### 4.1 Traditional invasive analysis

Most clinical or research settings involve the use of invasive procedures to analyze lactate and glucose. The lactate and glucose levels are determined by running invasively sampled blood specimens through analyzers. Typically, lactate is measured in fully automated clinical chemistry analyzers in pathology departments using whole anticoagulated blood samples.

Blood samples are typically drawn from arteries, veins, fingertips, or earlobe capillaries. Arterial sampling is less preferred owing to the need to access deeper blood vessels and consequent risks (Dassonville et al., 1998). Venous samples are collected via intravenous access lines (Crapnell et al., 2021). Capillary blood collected from fingertips or earlobes is often analyzed using lactate analyzers based on enzyme amperometric sensor chip systems, allowing measurements with small sample volumes (20 μL). Lactate levels tend to be higher in samples drawn from fingertip samples than those drawn from earlobe samples (Forsyth and Farrally, 2000). In sports settings, individual measurements may need to be repeated at rest, during exercise, and post-exercise, depending on the research procedure and objective to observe changes in lactate levels.

Yellow spring Instruments (YSI) analyzers are commercial laboratory analyzers designed for measuring lactate and glucose in blood, plasma, and serum. These analyzers utilize two interference-selective membranes with immobilized substrate-specific enzymes. The membranes are connected to platinum electrodes, which allow for highly specific and accurate measurements. Biosen analyzers are available in two variants: single-channel and dual-channel glucose systems. These devices employ specialized chip sensor technology to achieve highly specific measurements (Moradi et al., 2024).

The traditional method of measuring blood glucose and lactate levels involves pricking the fingertip or earlobe with a special needle to draw blood, so the process can cause some discomfort and stress (Daboss et al., 2022). The needles and solutions used for sampling are consumable, entailing high maintenance costs. While feasible in research settings, this approach has limitations in sports environments. A significant concern is the need to momentarily stop the activity for sampling during certain sports, which can disrupt athlete performance (Shitanda et al., 2023). This interruption may lead to differences between physiological responses measured during the testing and those that occur in actual competition scenarios.

### 4.2 Noninvasive analytical technique

**FIGURE 4:**
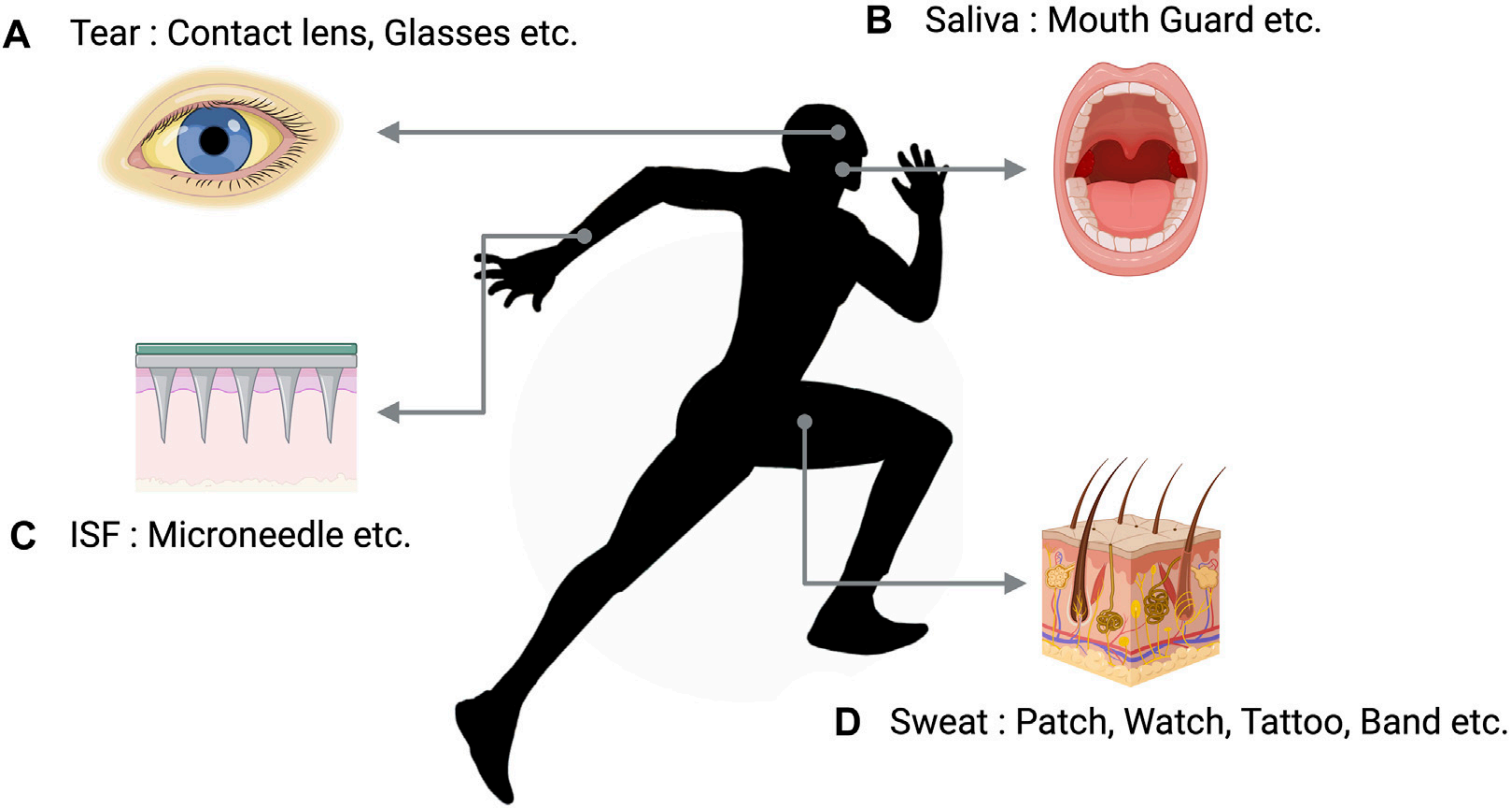
Body fluids used for non-invasive biomarker monitoring. **(A)** Instruments designed to analyze tears are created in the shape of lenses or spectacles. **(B)** Devices that analyze Saliva were developed in the form of a mouthguard. **(C)** ISF analysis devices consist of a microneedle that is affixed to the skin in order to monitor biomarkers. **(D)** Sweat analysis devices may manifest as various wearable technologies that incorporate biosensors. Recreated with BioRender.com.

In recent years, noninvasive technologies for collecting and analyzing biological fluids have been extensively researched to overcome these limitations and realize real-time monitoring. Tears, saliva, interstitial fluid (ISF), and sweat are typical bodily fluids that can be analyzed for metabolites in a completely noninvasive manner and thus suitable for use in clinical or sports settings (Moradi et al., 2024) (Figure 4).

Tears allow for the monitoring of health and physiological parameters in clinical or sports settings simply through the wearing of contact lenses. Yao et al. (2011) reported that contact lenses with an integrated amperometric glucose sensor are capable of detecting glucose at concentrations below 0.01 mM with rapid response (20 s), high sensitivity (240 μAcm^−2^mM^−1^), and good reproducibility. Amorphous indium gallium oxide field-effect transistor is a promising technology that can act as a transducer for detecting glucose *in vitro* and can be embedded in contact lenses for glucose monitoring via tears (Du et al., 2016; Gao et al., 2018). However, continuous extraction of tears for real-time monitoring is not feasible.

Saliva can be conveniently and continuously sampled by integrating sensors into mouthguards, and due to the correlation between biological biomarkers collected from saliva and those from the blood, saliva is considered a promising medium for noninvasive monitoring (Kim et al., 2014). Kim et al. (2014) described a mouthguard biosensor for continuous lactate detection in undiluted saliva samples (Gao et al., 2018). However, saliva is prone to contamination from factors such as food intake.

Chang et al. (2022) introduced a noninvasive wearable device technology capable of real-time monitoring of ISF glucose. While ISF demonstrates a good correlation with blood physiological biomarkers, most technologies, with a few exceptions, require minimally invasive procedures (Crapnell et al., 2021). These reasons may be why measuring biological biomarkers in sweat, which is relatively easier to collect and less prone to contamination-induced data errors, is being actively researched. Recent advancements suggest the feasibility of developing wearable devices capable of measuring concentrations of glucose, lactate, sodium ions, and potassium ions in sweat produced during exercise (Gao et al., 2018). This technology could enable regular glucose monitoring in patients with diabetes mellitus without resorting to traditional invasive blood glucose testing methods. Recent studies propose using biological fluid-based glucose detection technologies that creatively combine wearable devices with noninvasive glucose monitoring to enhance diabetes management. Typically, glucose concentrations are lower in sweat than in blood. Further, during exercise, the glucose concentration in sweat initially rises and falls with sustained activity. Clear elucidation of the relationship between blood and sweat glucose concentrations is crucial for sweat to be considered a viable alternative for continuous glucose monitoring (CGM).

The concentration of lactate in sweat is usually higher than that in blood, and precise observation within the range determining the LT (<4 mM) is essential for its application in sports. While blood lactate levels can remain stable or decrease with constant exercise power output over time, sweat lactate level tends to continuously increase. Therefore, the correlation between blood and sweat lactate levels needs to be further investigated, and technologies capable of producing reliable results at varying exercise intensities and durations need to be developed.

### 4.3 Continuous glucose monitoring using ISF

ISF is the most widely used body fluid for monitoring patients with diabetes. The U.S. Food and Drug Administration (FDA)-approved ISF glucose-based CGM technologies utilize electrochemical methods, where currents generated when ISF glucose is broken down by glucose-degrading enzymes such as glucose oxidase (GOx) are measured using microneedle sensors (Gao et al., 2018). An alternative method, fluorescence glucose sensing, can offer more accurate monitoring than electrochemical approaches, but some fluorescing chemicals used in this method can be toxic and thus have safety concerns (Klonoff, 2012). Nemaura Medical (UK) has released a wearable device using reverse iontophoresis to measure glucose noninvasively in the ISF. This product has received the Conformité Européene (CE) mark, a certification as reputable as the FDA in Europe (Gao et al., 2018). There is a strong correlation between glucose concentrations in ISF and blood (Kim et al., 2019). Simple devices enabling real-time glucose monitoring during training or competitions could allow for individualized and practical athlete management. The U.S. Women’s Olympic Cycling Team already used ISF-based CGM technology at the 2012 London Olympics. Nevertheless, most studies still utilize minimally invasive procedures to measure ISF glucose (Gao et al., 2018).

### 4.4 Benefits and limitations of traditional and noninvasive methods in sports

The traditional method of lactate measurement through blood sampling is well-established, offering proven accuracy and extensive research on protocols and practical applications for sports settings. Blood lactate concentration provides a sensitive indicator of physiological changes associated with exercise intensity. However, blood sampling is non-continuous, as it restricts the patient’s movement, which can lead to significant discrepancies between the real competition and the actual measurement time.

Conversely, noninvasive methods reduce discomfort for the patient and enable continuous, real-time monitoring. However, each body fluid has limitations (e.g., tears are challenging to collect, saliva is prone to contamination, and sweat has higher concentrations than blood). The technology is relatively new, necessitating further research. With more studies improving technology for consistent fluid collection in varying environments, shedding light on the differences with blood lactate, or establishing new standards related to sports performance, the technology will potentially replace traditional lactate measurement and contribute to enhancing sports performance.

## 5 Industry trends and developments

### 5.1 Athlete management system

Many attempts have been made to introduce technologies in sports that enable real-time monitoring of athletes’ activities and biometric data through wearable devices to enhance performance and provide systematic coaching (Li et al., 2016; Moore and Willy, 2019). The most common forms of wearable devices include biosensors integrated into smartwatches, bands that can be worn on arms and legs, and patches that can be placed on the desired body part. These wearables can be used not only for athlete management but also for monitoring the fitness and wellness of non-athletes as well as for real-time diagnosis of metabolic and cardiovascular diseases.

Athlete management systems that evaluate and manage athletes’ performance based on biometric data and real-time activity information are offered by many sports-related companies. Orreco (Ireland) collects biometric information through invasive methods and provides solutions based on this data. Companies such as Kinduct (Canada) and Edge10 (United Kingdom) have systems that simultaneously analyze biometric data and activity information, but they collect data via an external service. Obelab (South Korea) has launched a product that estimates blood lactate levels using real-time muscle oxygen saturation (SmO_2_) data measured by a wearable device worn on the thigh and offers individualized training programs. Garmin (United States of America) is researching algorithms to estimate LT based on the HR measured by smartwatches, although the smartwatches do not directly measure blood lactate.

Displaying real-time biometric data is as important as measuring them. For athletes or coaches to immediately apply the given data in training, they must have access to the monitoring data as needed without interrupting the training. Devices that analyze pace, HR, exertion, and oxygen saturation based on GPS data through smartwatches have become popular among recreational runners. Companies such as Solos (United States of America) and Everysight (Israel) launched products that display real-time information on lenses integrated into glasses, and Form (Canada) released smart swim goggles. While some have attempted to integrate sensors that collect biometric data into glasses-style devices, most rely on external devices to transmit and display measured data. This allows athletes to conveniently check simplified data in real time during exercise, and detailed information is stored on smartphones or tablets for post-training analysis.

### 5.2 Noninvasive glucose/lactate monitoring technology

Many invasive or minimally invasive sensors have been commercialized for patient monitoring. However, many companies are researching noninvasive fluid collection and analysis technologies, which could have the potential to replace the current invasive methods and become more mainstream.

Pkvitality (France) is developing a technology where a sensor is embedded in the back of a watch to measure glucose and lactate every 5 minutes and is aiming to launch the product in 2024. The company is currently conducting clinical trials for medical device certification. Abbott (United States of America) is in the research and development phase of integrating lactate measurement into its already commercialized glucose management systems. Lingo is a convenient wearable sensor attached to the back of the arm. Quantum Operations (Japan) is developing technology to measure glucose in the bloodstream through the skin using spectral detection techniques. Samsung (South Korea) is collaborating with the Massachusetts Institute of Technology (MIT) to develop glucose monitoring technology using Raman spectroscopy. This technology is anticipated to be featured in Samsung’s new generation of smartwatches, but it has not been implemented yet. Apple (United States) has been attempting to develop a glucose monitoring sensor for over 12 years but has failed to produce significant data. Currently, the company is focusing on research for sensing algorithms and accuracy. Noviosense (Netherlands) is working on a technology to measure glucose through tears using a device placed inside the lower eyelid.

Additionally, research teams from UCLA/Stanford (United States), HME Square (South Korea), Bioptx™ (United States), Verily (United States), and Cygnus (United States) are either in the research phase or have halted development for noninvasive real-time monitoring wearable device technologies. The significant investment and involvement of many companies and research institutions in this field attest to the growing demand and need for such technology in the field (Tables 1, 2).

**TABLE 1:** Current progress in the development of noninvasive glucose monitoring technologies.

Company (country)	Commercialization	Stage of development	Current progress
Pkvitality (France)	X (Targeting 2024)	Research	• Research initiated in 2016 and is currently in progress; clinical trial is ongoing. Commercialization expected in 2024
			• K’apsul is clicked on the back of the watch and replaced every 7 days. Measurements taken every 5 min
			• Anticipated price: K’Watch $199 (K’apsul Glucose sensors $99.90/month)
			• Sensing range beyond glucose: Activity tracking (steps, active minutes, and calories)- Heart rate- Sleep quality- Alarms: General alarms and alarms for hypoglycemia and hyperglycemia
Novio Sense (Netherlands)	X	Research	• Placed under the lower eyelid to measure blood glucose in tears
			• Hydrogel enzyme (glucose oxidase) measures current via a small, flexible, coil-shaped electrode (2-cm long)
			• Since being founded in 2012, there is ongoing Research and Development (R&D); however, specific details and results are not publicly disclosed
Quantum Operations (Japan)	X	Research	• Development underway, including continuous glucose monitoring (CGM) feature
			• Measures glucose in human blood flow through the skin using spectrum detection technology
			• Development underway, including technology for monitoring HR and blood oxygen saturation changes; however, details are not disclosed
Bioptx™ (United States)	X	Research paused	• Technology integrating proprietary infrared (IR) laser detection, photoplethysmography (PPG) sensors, and proprietary algorithms is constructed using software
			•Developing pulse and oxygen saturation measurement technology in smartwatches using skin-illuminating LEDs
Verily (United States)	X	Research paused	• Suspension of development of IEEE spectrum smart contact lenses (2018)
			• Developing technology for tear glucose monitoring
			•Alphabet/Google subsidiary
			• Lack of proven correlation between tear and blood glucose levels according to medical device standards
Apple (United States)	X	Research	• Apple has been developing glucose monitoring sensors for 12 years but has failed to produce significant clinical data (2023 Bloomberg)
			• Prepared to equip Applewatch 9 with glucose monitoring technology but halted by technological limitations
			• Conducting additional research on sensing algorithm and accuracy of sensors
			• Commercialization projected to take between 3 and 7 years
Samsung (Republic of Korea)	X	Research	• Developing innovative, noninvasive blood glucose monitoring using Raman spectroscopy developed jointly with MIT
			• Commercialization of noninvasive CGM technology estimated to require hundreds of millions or over a billion dollars (DexCom)
			• Expectations for Samsung’s Galaxy Watch to feature glucose monitoring sensors unmet in current models
Cygnus (United States)	X	Research paused	• Developing sensors enabling up to 12 h of CGM (GlucoWatch)
			• Developing technology for directly measuring glucose concentration using electrochemical sensing technology by attracting interstitial fluid containing glucose molecules to the skin surface with the currents of the GlucoWatch
			• The current required to extract glucose caused skin irritation, redness, burns, and blisters. GlucoWatch could not accurately detect rapid glucose changes
HME Square (Republic of Korea)	X	Research	• Founded in 2020. Wearable noninvasive CGM using MEMS-based photoacoustic technology
			• Photoacoustic technology, MEMS sensors, and deep learning algorithms
			• Details of development not disclosed

**TABLE 2:** Current progress in the development of noninvasive lactate monitoring technologies.

Company (country)	Commercialization	Stage of development	Current progress
Pkvitality (France)	X (Targeting 2024)	Research	• Research initiated in 2016 and is currently in progress; clinical trial is ongoing. Anticipated commercialization in 2024
			• K’apsul is clicked on the back of the watch, replaced every 7 days. Measurements taken every 5 min
			• Continuous monitoring in 30-day cycles
			• Accuracy not precisely mentioned; clinical trial underway to obtain medical device certification, and only mentions the technology should be accurate for validation
			• Anticipated price: K’Watch $199 (K’apsul Glucose sensors $99.90/month)
			* Mentions that the price may vary depending on region and taxes at the time of launch
			• Features available
			- Time and date
			- Activity tracking (steps, active minutes, calories)
			- Heart rate
			- Sleep quality
			- Alarms: general alarms and alarms for hypoglycemia and hyperglycemia
UCLA/Stanford (United States)	X	Research	• Published research on a smartwatch technology for determining body’s drug concentration by analyzing sweat on PNAS
			• Published research regarding prototype development in 2016
			• Laboratory prototype developed, but no details on commercialization are available
Abbott (United States)	X	Research	• Lingo is an expansion of Abbott’s flagship product, the Freestyle Libre glucose management device
			• Product design resembles the Freestyle Libre, featuring a circular sensor that minimizes discomfort and is attached to the back of the arm
			• Currently in R&D phase (announced at CES 2022)
Garmin (United States)	X	Research	• Could not develop technology for directly monitoring lactate in sweat
			• Lactate threshold indirectly measured using Garmin’s heart rate monitor HRM-Dual sensor and algorithmic calculations

## 6 Sweat glucose/lactate biosensors

### 6.1 Enzymes

The interest and investment in developing noninvasive technologies for lactate measurement have been increasing significantly. Glucose is broken down into the intermediate metabolite pyruvate. Under aerobic conditions, pyruvate is converted into acetyl coenzyme A by pyruvate dehydrogenase (PDH) before entering the Krebs cycle. However, under anaerobic conditions, pyruvate is transformed into lactate by lactate dehydrogenase (LDH).

Two primary methods are used for lactate measurement: (1) using LDH and (2) using lactate oxidase (LOx) (Saha et al., 2022). The LDH method relies on spectrophotometric measurements of light absorption before and after adding LDH to the sample, and this reflects the amount of NADH formed as a result of lactate metabolism (Crapnell et al., 2021). The second method using LOx is the one used in most devices. In this approach, lactate reacts with LOx to form hydrogen peroxide (H_2_O_2_), and the resulting current is measured using amperometry. Although there are other methods involving lactate monooxygenase, flavocytochrome b2, and cytochrome b2, these methods are less commonly employed.

LOx is preferred to LDH in noninvasive monitoring devices because the latter, while accurate, requires an additional coenzyme (NAD^+^). LOx oxidizes L-lactate to pyruvate through the reduction of its cofactor, flavin mononucleotide (FMN). Designed to be less sensitive to oxygen, LOx essentially utilizes artificial electron acceptors to reoxidize FMN. The reduced artificial electron acceptor can transfer electrons between LOx and the electrode. However, a limitation of the LOx method is that it can produce erroneous readings due to glycolate, a metabolite of ethylene glycol (Crapnell et al., 2021).

### 6.2 Biosensors

**FIGURE 5:**
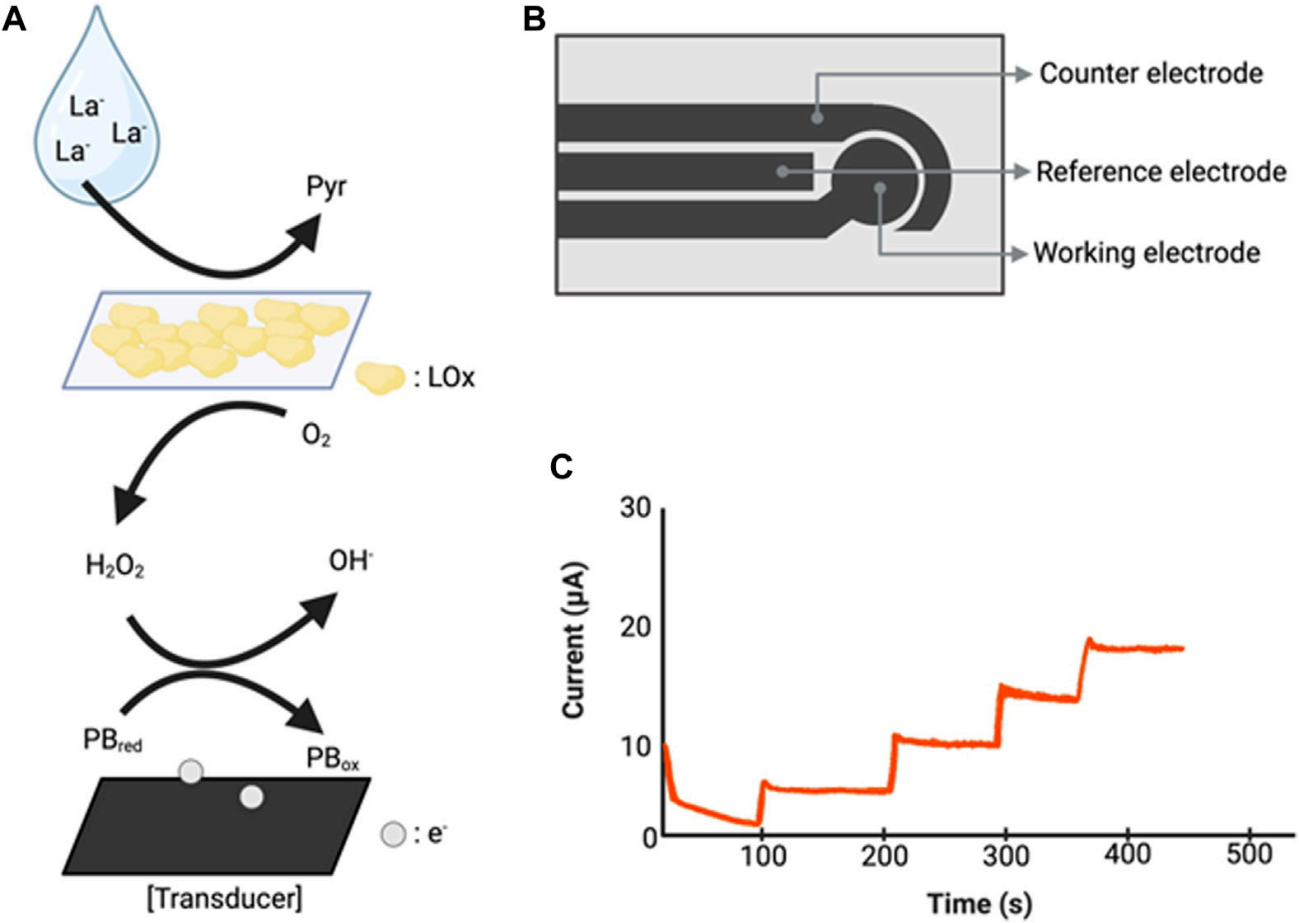
Illustration of biosensors used in sweat lactate monitoring technologies. **(A)** Lactate detection mechanism operating at a working electrode. Lactate reacts with the lactate oxidase of the sensor to produce pyruvate and H_2_O_2_. H_2_O_2_ reacts with the Prussian blue transducer and releases electrons (Xuan et al., 2023a). **(B)** Illustration of sensor chip (Seki et al., 2021). **(C)** A hypothetical graph showing the amperometric reaction according to changes in lactate concentration. La^−^: Lactate, Pyr: Pyruvate, LOx: lactate oxidase, PB_red_: Prussian blue reduced, PB_ox_: Prussian blue oxidized, e^−^: electron. Created with BioRender.com.

To detect signs of disease and prevent progression to advanced disease, technology capable of sensitively monitoring even minor physiological changes is essential. This sensitivity is crucial in sports settings as well, where monitoring athletes’ training intensity is key to planning schedules and preventing injuries. In response to these needs, there has been significant progress over the past decade in developing wearable devices that integrate sensors for analyzing fluid data collected through the skin using wristwatches, headbands, and clothes (Khan et al., 2022; Wang et al., 2022; Konno and Kudo, 2023) (Figure 5).

For noninvasive monitoring, efficient sampling of the analyte, precise binding between the analyte and its receptor, and accurate signal transmission of the energy generated during the receptor-analyte reaction are crucial aspects (Moradi et al., 2024). Additionally, such sensor technologies need to be compatible with wearable devices such that they do not hinder the performance of the wearer during physical activities. The electrochemical approach is the most extensively researched method for transmitting lactate signals detected in sweat. Electrochemical sensors measure lactate by detecting changes in electrical potential generated by enzymatic oxidation-reduction reactions. These devices are becoming increasingly compact, allowing easy integration with wearable devices, precise detection, low detection limits, and suitability for long-term use. Despite the relative ease of collecting sweat in sports settings compared to collecting sweat from patients with limited mobility in clinical situations (Ghaffari et al., 2021), there are several challenges to be addressed.

The technology for collecting sweat must maintain qualitative and quantitative performance for analysis in conditions with both low perspiration (e.g., resting or cold environments) and high perspiration (e.g., exercise or in humid environments). Additionally, aquatic conditions such as swimming and diving must also be considered. Even with efforts to induce high perspiration, there is often a delay between the start of physical activity and the onset of sweating. In a study by Imani et al. (2016), early levels could not be measured in intense cycling sprints lasting 15–30 min due to insufficient sweating. However, in a study by Martín et al. (2017), the microfluidic system directly transferred sweat from the glands to an 8.72 μL sample chamber, and the chamber was filled in 13.4 min by targeting four sweat glands producing sweat at 20 nL/min. Some studies employ passive methods to stimulate and maintain consistent sweating. These methods include the use of cholinergic agonists, such as pilocarpine (Baker and Wolfe, 2020). Pilocarpine stimulation does not affect sweat lactate concentration (Derbyshire et al., 2012), can increase resting sweat rates by 5–10 times, and induces more sweating during exercise. However, this step requires additional power consumption, necessitating a larger power supply unit that would increase the product’s size and weight. To counter these disadvantages, Saha et al. (2022) examined the effectiveness of a lactate monitoring platform that allowed the collection of sweat over extended periods using hydrogels for osmotic sweat extraction and paper microfluidic channels for sample evaporation. They reported sweat lactate concentrations of approximately 2–3 mM in sweat collected for up to 100 min at rest, 7–9 mM in sweat collected during 1 h of moderate-intensity exercise, and 10–12 mM in sweat collected during 30 min of high-intensity exercise. Komkova et al. (2022) used two high-accuracy, low-power wearable controllers (UMKA) on the same muscle to independently verify sweating intensity and lactate concentration and confirmed that sweating intensity and lactate concentration are independent of each other. They found that this is the major cause of real-time monitoring errors based on electrochemical sensors relying on flow. Integrating such sweat induction technologies could address the challenges of limited sweat collection due to varying sweating rates in different environmental conditions. Moreover, it can circumvent the oversight of early lactate monitoring during physical activity (Imani et al., 2016).

Given their design, wearable devices must be attached to the body using adhesives or bands, which may lead to detachment from the skin or alteration of the skin surface during vigorous competition, potentially resulting in measurement inaccuracies. Jia et al. (2013) introduced an electrochemical tattoo biosensor that minimized restrictions posed by the appearance of the wear site and maintained high stability even in the presence of skin deformations during movement. This sensor used LOx-based amperometric detection and employed a CNT/TTF composite to facilitate effective electron transfer and address potential electroactive interferences. Additionally, the sensor successfully detected LT, demonstrating the potential for sports performance monitoring. Wang et al. (2022) developed a tiny, 1.5 mm × 1.5 mm MS02 chip capable of measuring glucose, lactate, Na^+^, and K^+^. Its small size facilitated wearability during physical activities and has limited sample volumes. The participants wore the MS02 chip on the forehead during a 10-min cycling session, and the detection ranges were 0–300 μM for glucose and 5–25 mM for lactate. The data collected by the chip were transmitted to a separate application for analysis. These two studies demonstrate the potential to avoid errors arising from device detachment from the skin by using materials that can flexibly change shape according to skin deformation or sensors of small size that are minimally affected by deformation. When using a biosensor, it is equally important to maintain accurate results over a long period of time as it is to accurately measure the concentration of biomarker. The sensor introduced by Jia et al. (2013) had a highly linear response in the 1–20 mM range, with high stability over 8 h of use. Regarding the biosensor’s shelf-life, the sensor’s sensitivity decreased by less than 10% after 5 months of storage. Xuan et al. (2023a) developed sensors unaffected by pH over long-term use and successfully measured sweat lactate data in canoeists and cyclists. Shitanda et al. (2023) addressed the issue of unstable sensor responses and monitoring errors due to air bubbles trapped in conventional microfluidic channels by introducing a bubble-trapping region in the channel, thus mitigating the effects of air bubbles.

Colorimetric methods offer a simpler structure than devices based on electrochemical techniques, allowing more intuitive monitoring of lactate measurements. Electrochemical methods enable real-time monitoring data to be transmitted to external display devices, such as tablet computers, allowing coaches or managers to observe the athlete’s physiological changes. Furthermore, compared to colorimetric methods, accuracy and detail are superior. However, colorimetric approaches offer the simplest way for athletes to assess their condition with minimal interruption to their training. Analytes such as AnNP used in colorimetry change color based on the concentration of the target compound, enabling users to easily identify their status (Moradi et al., 2024). Kim et al. (2022) introduced a technology where hydrogen peroxide produced by the reaction between lactate with LOx reduced polyaniline (PAni) in the form of emeraldine base (EB) to emeraldine salt (ES) to monitor lactate levels based on the color reflecting this change. Each state changes with pH levels, allowing visual monitoring of pH as well. Promphet et al. (2019) developed a fiber-based colorimetric sensor capable of simultaneously detecting pH and lactate in sweat by depositing three different layers on cotton fabric: chitosan, sodium carboxymethyl cellulose, and indicator dyes or reagents for lactate analysis. The sensor visualizes concentrations, where color changes from red to blue with increasing pH (1–14), and various intensities of purple develop according to the lactate concentration (0–25 mM). Koh et al. (2016) examined a device that measures not only lactate but also total sweat loss, pH, chloride, and glucose concentrations through colorimetric detection. LDH was used for lactate detection, showing color changes across the range of 1.5–100 mM. For glucose detection, H_2_O_2_ produced by the reaction between glucose and GOx oxidized iodide to iodine, the color change from yellow to brown became more prominent as the glucose concentration increased. While sweat glucose concentrations are lower than blood glucose concentrations, the range detected by this device was sufficient for diagnosing hyperglycemia.

### 6.3 Comparison of noninvasive lactate monitoring biosensors

Table 3 highlights studies that have validated the performance of biosensors during exercise. Electrochemical methods are the most commonly utilized in this field. Colorimetric approaches have been less extensively researched, as even minor differences of 0.five to one mM can alter result interpretation for lactate evaluations for sports performance. Thus, electrochemical methods may be preferred for their higher precision.

**TABLE 3:** Comparative analysis of noninvasive sweat lactate monitoring biosensors in sports performance studies.

Study References	Biosensor method	Exercise Modalities Tested	Collection Sites	Measurement Range (mM)	Enzyme	Key Findings
Jia et al. (2013)	Electrochemical	SC	Single site (Arm)	1 to 20	LOx	Using electrochemical biosensors, a flexible printed temporary-transfer tattoo that adapts to the wearer’s skin
Imani et al. (2016)	Electrochemical	SC	Single site (Chest)	0 to 28	LOx	a skin-worn wearable hybrid sensing system that offers simultaneous real-time monitoring of a biochemical and an electrophysiological signal
Martín et al. (2017)	Electrochemical	SC	Single site (Back)	4 to 20	LOx	Highlighted the need for accuracy in the LT test range and the comparison between blood and sweat lactate levels
Karpova et al. (2020)	Electrochemical	CE	Two different sites (Thigh, Arm)	N/A	LOx	Contributed to guidelines for sensor placement depending on the sport by analyzing sweat from multiple sites
Mao et al. (2020)	Electrochemical	SC	Single site (Knee joint)	N/A	LOx	By actively outputting piezoelectric signals, body movements and physiological information can be detected quickly and sensitively
Klous et al. (2021)	Electrochemical	SC	Two different sites (Arm, Back)	N/A	-	Addressed the variation in sweat lactate concentration by site, aiding in sensor placement strategies
Seki et al. (2021)	Electrochemical	CE	Two different sites (Arm, Forehead)	0 to 5	LOx	Demonstrated the importance of considering sweat collection site in sensor design and data interpretation
Daboss et al. (2022)	Electrochemical	Squat	Two different sites (Thigh, Arm)	0.5 to 100	LOx	Supported the relevance of dual-site sweat collection for comprehensive lactate monitoring
Khan et al. (2022)	Electrochemical	running	Two different sites (Chest, Forehead)	0.4 to 1.3	LOx	Highlighted site-dependent sweat lactate variations, emphasizing the need for site-specific monitoring guidelines
Komkova et al. (2022)	Electrochemical	running	Single site (Thigh)	10 to 30	LOx	Emphasized the significance of measuring within the physiological change range during exercise
Saha et al. (2022)	Electrochemical	CE	Single site (Forearm)	0 to 15	LOx	Combining hydrogels for osmotic sweat extraction and paper microfluidic channels to promote sweat transport
Wang et al. (2022)	Electrochemical	SC	Single site (Forehead)	5 to 25	LOx	Suggested that sweat lactate concentrations might be higher than blood concentrations, indicating a need for clear correlation verification
Okawara et al. (2023)	Electrochemical	CE	Single site (Arm)	0 to 5	-	Study whether VT and blood LT can be assessed through sweat lactate monitoring
Shitanda et al. (2023)	Electrochemical	SC	Single site (Back)	1 to 50	LOx	Development of a lactate sensor with microchannels to overcome the air bubble problem that prevents measurement of lactate levels in sweat
Xuan et al. (2023a)	Electrochemical	CE, KE	Two different sites (Thigh, Back)	1 to 20	LOx	Verified performance in diverse exercises, suggesting the versatility of electrochemical methods
Koh et al. (2016)	Colorimetric	CE, road cycling	Two different sites (Arm, Back)	1.5 to 100	LDH	Demonstrated feasibility across environments and situations, emphasizing the practicality of sweat lactate monitoring
Promphet et al. (2019)	Colorimetric	running	Single site (Upper body)	0 to 25	LOx	Success in real-time exercise monitoring, highlighting colorimetric’s visibility advantage for athletes

CE, cycle ergometer; SC, stationary cycling; KE, kayaking ergometer; LOx, lactate oxidase; LDH, lactate dehydrogenase; mM, millimole.

Running and cycling were the primary exercises used for exercise evaluation. This could be due to their widespread use in studies on traditional lactate measurement. Xuan et al. (2023a) validated performance in cycling and kayaking ergometers. Moreover, Koh et al. (2016) validated performance in both indoor and road cycling, demonstrating the potential for using sweat lactate monitoring in research and games (or training) without restrictions.

Once the performance of these biosensors is validated for sports traditionally used in lactate-related research, such as marathon, rowing, combat sports, and ball sports, as well as in areas in which research was challenging due to the limitations of the traditional methods, the benefits of sweat lactate monitoring would become more evident.

Many studies collected sweat samples from one area of the body, but Karpova et al. (2020), Klous et al. (2021), Seki et al. (2021), Daboss et al. (2022), Khan et al. (2022), Xuan et al. (2023a) and Koh et al. (2016) collected sweat samples from two different areas of the body. As previously mentioned, sweat lactate concentration varies across the site of collection. Therefore, these studies may provide guidelines on sensor placement specific to each type of sport.

The range of linear detection of sweat lactate concentrations varied widely from 0 mM to 100 mM. Accuracy within the 1–5 mM is particularly important for exercise performance evaluations (especially LT test), as this is the reference range for physiological changes during exercise. Martín et al. (2017), Komkova et al. (2022), and Wang et al. (2022) showed measurements beyond this range. However, this may be attributed to the fact that lactate concentrations tend to run higher in sweat than in blood. Thus, the relationship between blood and sweat lactate levels should be clarified.

Koh et al. (2016) and Promphet et al. (2019) successfully measured lactates during exercise using the colorimetric approach. Although electrochemical methods also allow real-time monitoring, the visual indications provided by a colorimetric method could be more user-friendly for the athletes to monitor their physiological changes in real-time. Most studies used LOx. As previously mentioned, this may be attributed to the advantages of LOx over other enzymes, such as LDH.

## 7 Relationship between blood lactate and sweat lactate

Determining the correlation between metabolites measured in blood and sweat is a crucial issue in the field that must be addressed before implementing sweat lactate monitoring using wearable devices. The rate of sweat production, collection site, and method can all impact this correlation, highlighting the need for detailed and systematic research.

Xuan et al. (2023a) analyzed the correlation between blood and sweat (back and thigh) lactate levels during progressively intense cycling on an ergometer and revealed a significant correlation between blood and thigh sweat lactate concentrations during cycling. Karpova et al. (2020) studied 10 adult men (age, 18–35 years) and reported a positive correlation between variability in blood and sweat (arm and thigh) lactate levels during progressively intense cycling, based on which they argued that variability should be the focus, as opposed to lactate concentrations in the blood and sweat. Most participants showed an increase in thigh sweat lactate concentrations during exercise, but not arm sweat lactate concentration, and some even showed a decrease. This may be attributed to the nature of cycling.

In a study that revealed a correlation between blood and sweat (back) lactate levels during progressively intense aero-bike exercise in men in their 40 s, Shitanda et al. (2023) demonstrated that blood lactate showed changes at the onset of exercise, while sweat lactate data was observable only 1,600 s after starting the exercise. This finding can be attributed to the time required to collect sufficient sweat for detection. Similar limitations have been observed in several other studies, albeit varying, depending on the sensor technology, underscoring the need to consider these characteristics when interpreting real-time sweat monitoring data.

Green et al. (2000) reported that increments in blood lactate levels were not correlated with changes in sweat lactate levels in a study of participants cycling at 40% $\dot{V}O_{2}\max$ for 30 min and participants performing interval cycling trials at a two-fold higher load. Klous et al. (2021) analyzed the correlation between six biomarkers in blood and sweat (arm and back) in 12 trained adults (age, 21–29 years) during cycling at 60%, 70%, and 80% of the HRmax. They found no significant correlations at any exercise intensity, though a significant correlation was observed between blood and upper back sweat glucose at 70% HRmax.

While many studies have observed a high correlation between blood and sweat metabolites (Xuan et al., 2023b; Rabost-Garcia et al., 2023), others have claimed no such correlation (Lamont, 1987). However, the studies that reported no correlation had small sample sizes, employed non-standard methods of sample collection and analysis, did not distinguish between active and latent muscles, and had imprecise research designs (Karpova et al., 2020). Nevertheless, the lack of consistency in the ratios of biomarkers in sweat and blood in many studies might be due to relatively scant data (Xuan et al., 2023b). However, the clear difference in metabolite amounts in sweat (5–40 mmol/L) and blood (0.5–25 mmol/L) (Baker and Wolfe, 2020) suggests that correlation and regression analyses specific to the situation and environment are needed (Rabost-Garcia et al., 2023). Moreover, given the differences in metabolite concentrations based on the site of sweat collection, further research is needed to examine concentrations depending on the type of exercise and placement of the wearable device on the body.

## 8 Conclusion

This review highlights that non-invasive lactate monitoring through sweat during exercise has been extensively researched, and relevant devices are close to being commercialized. This review sheds light on the potential of sweat in offering a more stable and convenient means for lactate measurement than other bodily fluids through wearable devices that provide real-time data. For the successful commercialization of noninvasive lactate monitoring devices, several key challenges must be addressed. Particularly, the focus should be on improving device accuracy and reliability. Current research on the quantitative relationship between lactate levels in blood and sweat remains inadequate. As there is clearly a gap between the concentrations in blood and sweat, sophisticated algorithms that can accurately estimate blood metabolite levels from sweat measurements are needed. Continuous research is needed to determine the precision of measurements based on lactate in sweat induced by exercise, to examine whether data can be corrected in real-time when the lactate level decreases with increasing sweat volume over time, and to address the limitations posed by the differences in concentrations across body sites. While the majority of the studies are focused on estimating biomarker concentrations in sweat, the practical aspects, such as whether these new methods can fully replace traditional blood lactate measurement protocols and the reliability of real-time monitoring data during training and competitive scenarios also need to be explored. Additionally, safeguarding data security is an essential consideration when sharing the data of elite athletes via cloud systems.

Future research should aim at refining device performance and delve deeper into sophisticated data analysis and interpretation techniques. In addition, more field experiments are needed to expand the applicability of this technology, which requires continued interaction between the sports and technical fields. Such endeavors will help enhance athletic performance, aid in injury prevention, and optimize training periodization. Considering these challenges and competencies, the use of wearable devices for monitoring sweat lactate is paving the way for innovative technology in sports science, underscoring the need for continued research and progress in this burgeoning field.
